# A meta-analysis of the medium- and long-term effects of laparoscopic sleeve gastrectomy and laparoscopic Roux-en-Y gastric bypass

**DOI:** 10.1186/s12893-020-00695-x

**Published:** 2020-02-12

**Authors:** Lihu Gu, Xiaojing Huang, Shengnan Li, Danyi Mao, Zefeng Shen, Parikshit Asutosh Khadaroo, Derry Minyao Ng, Ping Chen

**Affiliations:** 1 Department of General Surgery, HwaMei Hospital, University of Chinese Academy of Sciences, Northwest Street 41, Haishu District, Ningbo, Zhejiang, 315010 China; 2 The Second Clinical Medical College, Zhejiang Chinese Medical University, Hangzhou, Zhejiang China; 3 Basic Medical College, Zhejiang Chinese Medical University, Hangzhou, Zhejiang China; 4 Department of General Surgery, Zhejiang University School of Medicine Sir Run Run Shaw Hospital, Hangzhou, Zhejiang China; 5 Monash University School of Public Health and Preventive Medicine, Melbourne, Australia; 6 Medical College of Ningbo University, Hangzhou, Zhejiang China

**Keywords:** Sleeve gastrectomy, Roux-en-Y gastric bypass, Effects, Meta-analysis

## Abstract

**Background:**

Laparoscopic Roux-en-Y gastric bypass (LRYGB) and laparoscopic sleeve gastrectomy (LSG) are two representative bariatric surgeries. This study aimed to compare the effects of the LSG and LRYGB based on high-quality analysis and massive amount of data.

**Methods:**

For this study databases of PubMed, Web of Science, EBSCO, Medline, and Cochrane Library were searched for articles published until January 2019 comparing the outcomes of LSG and LRYGB.

**Results:**

This study included 28 articles. Overall, 9038 patients (4597, LSG group; 4441, LRYGB group) were included. The remission rate of type 2 diabetes mellitus (T2DM) in the LRYGB group was superior to that in the LSG group at the 3-years follow-up. Five-year follow-up results showed that LRYGB had an advantage over LSG for the percentage of excess weight loss and remission of T2DM, hypertension, dyslipidemia, and abnormally low-density lipoprotein.

**Conclusions:**

In terms of the long-term effects of bariatric surgery, the effect of LRYGB was better than of LSG.

## Background

Worldwide, obesity not only seriously affects the external appearance but also causes various diseases, which threaten people’s health. The usual way to lose weight is through dieting or medication use, which reduces weight by 5 to 10%. However, the resulting weight loss is short term, leading to rebound weight gain. Nowadays, bariatric surgery is widely known, as it has long-lasting effectiveness [1, 2]. Meanwhile, it can also alleviate some complications [3].

Laparoscopic Roux-en-Y gastric bypass (LRYGB) and laparoscopic sleeve gastrectomy (LSG) are two representative bariatric surgeries. The origin of LRYGB can be traced back to about 50 years ago. As LRYGB has excellent effectiveness on alleviating obesity complications, including type 2 diabetes (T2DM), it is known as the gold standard surgery for obese patients [4–8]. However, impaired micronutrient absorption is more common after LRYGB [9–11]. Some studies had shown that patients treated with LRYGB were more likely to have vitamin B12 deficiency after surgery than patients treated with LSG, but there was no difference in the absorption of folic acid and the effect on serum iron was controversial [11–14].

Moreover, conclusions of studies that compared LRYGB with LSG in the remission of complications remained controversial. Some studies indicated that LRYGB was superior to LSG [5, 15, 16] in the remission of T2DM, while other studies suggested that the remission rates were similar in both groups [17]. Previous studies had shown an advantage for LRYGB in the remission of hypertension [18–20]. With these differences in results, this study aimed to scientifically compare the advantages of LRYGB and LSG based on high-quality analysis and massive amount of data.

## Methods

### Literature search

This meta-analysis was in line with the recommendations of Preferred Reporting Items for Systematic Reviews and Meta-Analyses (PRISMA) statement [21]. Electronic literature search was conducted from inception to January 2019 of various databases including PubMed, EMBASE, Cochrane library, Web of Science, and EBSCO. The following keywords were used: (“laparoscopic Roux-en-Y gastric bypass” OR “gastric bypass” OR “GB” OR “LRYGB”) AND (“laparoscopic sleeve gastrectomy” OR “sleeve gastrectomy” OR “SG” OR “LSG”). Two researchers separately performed the literature search and compared their results. Most of the articles were screened manually by scanning titles and abstracts. Then, through a further check, all initially included studies were downloaded finally.

### Inclusion and exclusion criteria

The primary outcomes of the studies included in the analysis were percentage of excess weight loss (%EWL) and remission rates of T2DM. The secondary outcomes of the studies were remission rates of hypertension and dyslipidemia. Published studies comparing the outcomes of LSG and LRYGB were considered potentially eligible. The follow-up time was at least 3 years. The exclusion criteria were as follows: (1) non-original article; (2) results did not include %EWL and remission rates of T2DM, hypertension, and dyslipidemia; and (3) no available medium-term (3-year) or long-term (5-year) original data or relevant outcome. Any unclear data were deleted decisively, and we made sure that all data were checked more than twice.

### Data extraction and quality assessment

After multiple inspections, a list of articles that finally met the inclusion criteria was created, and a reviewer extracted the following basic indicators from each article (Table 1): study country and year, sample size, follow-up time, number of patients who completed the final follow-up, and study type. The indicators of %EWL and remissions of T2DM, hypertension, and dyslipidemia in each article were extracted.

**Table 1:** Characteristics of studies included in the meta-analysis

Author, year	Country	No. of participants		Follow-up (year)	No. of remaining		Comorbidities remission (without medication)	Study type
		LSG	LRYGB		LSG	LRYGB		
Abbatini, 2010	Italy	20	16	3	20	16	FPG < 126 mg/dl, HbA1c < 6.5%	Retrospective
Ahmed, 2018	USA	59	57	7	27	26	NA	Prospective
Alexandrou, 2014	Greece	40	55	4	40	55	NA	Prospective
Dakour Aridi, 2018	Lebanon	400	175	5	87	118	NA	Retrospective
Boza, 2012	Chile	811	786	3	811	786	FPG < 126 mg/dl, HbA1c < 6.5%	Retrospective
Carandina, 2014	France	34	74	4	34	74	NA	Retrospective
Dogan, 2015	Netherlands	255	430	5	245	245	NA	Retrospective
Du, 2016	China	63	63	3	60	59	FPG < 5.6 mmol/l, HbA1c < 6%/BP < 120/80 mmHg	Retrospective
Climent, 2018	Spain	48	103	5	48	103	NA	Retrospective
Gonzalez-Heredia, 2016	USA	77	12	3	30	8	NA	Retrospective
Ignat, 2017	France	55	45	5	41	32	NA	RCT
Jammu, 2016	India	339	295	5	97	143	NA	Prospective
Jimenez, 2012	Spain	55	98	3	55	98	FPG < 126 mg/dl, HbA1c < 6.5% for at least 1 year	Prospective
Kim, 2019	Singapore	256	39	3	71	10	NA	Retrospective
Kaseja, 2014	Poland	33	41	3	33	41	NA	Prospective
Lager, 2018	USA	334	380	4	226	272	HbA1c < 6.5%/BP < 120/80 mmHg	Retrospective
Lee, 2015	China	519	519	5	116	218	NA	Retrospective
Leyba, 2014	Venezuela	42	75	5	27	47	HbA1c < 6%	Prospective
Perrone, 2017	Italy	162	142	5	162	142	NA	Retrospective
Peterli, 2018	Switzerland	112	113	5	101	104	FPG < 100 mg/dl, HbA1c < 6.0% at least 1 year	RCT
Rondelli, 2017	Italy	280	301	3	259	282	NA	Retrospective
Ruiz-Tovar, 2019	Spain	200	200	5	182	184	FPG < 110 mg/dl, HbA1c < 6.5%/BP < 135/85 mmHg/FPT < 200 mg/dl, TC < 200 mg/dl, HDL > 40 mg/dl	RCT
Salminen, 2018	Finland	121	119	5	98	95	FPG < 100 mg/dl, HbA1c < 6.0%/LDL < 115.8 mg/dl	RCT
Sepulveda, 2018	Chile	57	55	3	41	35	FPG < 100 mg/dl, HbA1c < 6.0%	Retrospective
Vidal, 2013	Spain	114	135	4	91	108	NA	Retrospective
Yang, 2015	China	32	32	3	28	27	HbA1c < 6.0%	RCT
Zhang, 2014	China	32	32	5	26	28	NA	RCT
Schauer, 2017	USA	47	49	5	47	49	HbA1c < 6.5%	RCT

*LSG* laparoscopic sleeve gastrectomy; *LRYGB* laparoscopic Roux-en-Y gastric bypass; *NA* no available; *FPG* fasting plasma glucose; *HbA1c* glycosylated hemoglobin; *BP* blood pressure; *FPT* fasting plasma triglycerides; *TC* total cholesterol; *LDL* low-density lipoprotein; *HDL* high-density lipoprotein; *RCT* Randomized clinical trial

The quality of included articles was evaluated using the Cochrane risk-of-bias tool and the Newcastle-Ottawa Quality Assessment Scale (NOS) checklist [22, 23]. According to the recommendations of the Cochrane manual, the risk of bias in randomized controlled trial (RCTs) is categorized as low risk, unclear risk, and high risk. Observational studies were assessed by the NOS. Each article was evaluated in three aspects as follows: object selection, inter-group comparability, and outcome measurement. Articles with scores < 6 were considered low-quality articles. Any contradiction between reviewers was resolved by the consensus of two authors and the third reviewer.

### Statistical analysis

RevMan 5.3 software was used to integrate statistical results. Weighted mean difference (WMD) was used to collect continuous variables, while odds ratios (OR) was used to analyze dichotomous variables. Heterogeneity was checked by Cochrane Collaboration’s risk of tool, chi-squared test, and I^2^ statistics and identified when *p* < 0.1 and I^2^ > 50%. If the results were heterogeneous, a random-effects model was used to calculate the combined effect size; otherwise, a fixed-effects model was used. The Stata 12.0 Software (Stata, College Station) was used to evaluate the sensitivity and publication bias of the studies. Publication bias was evaluated by Begg’s and Egger’s tests, and *p* < 0.05 was considered statistically significant. Begg’s and Egger’s tests of publication bias were not performed to analyze subgroups with less than 10 articles because of the low sensitivity of qualitative and quantitative tests. All statistical tests were two-sided, and *p* < 0.05 was considered statistically significant.

## Results

**Fig. 1:**
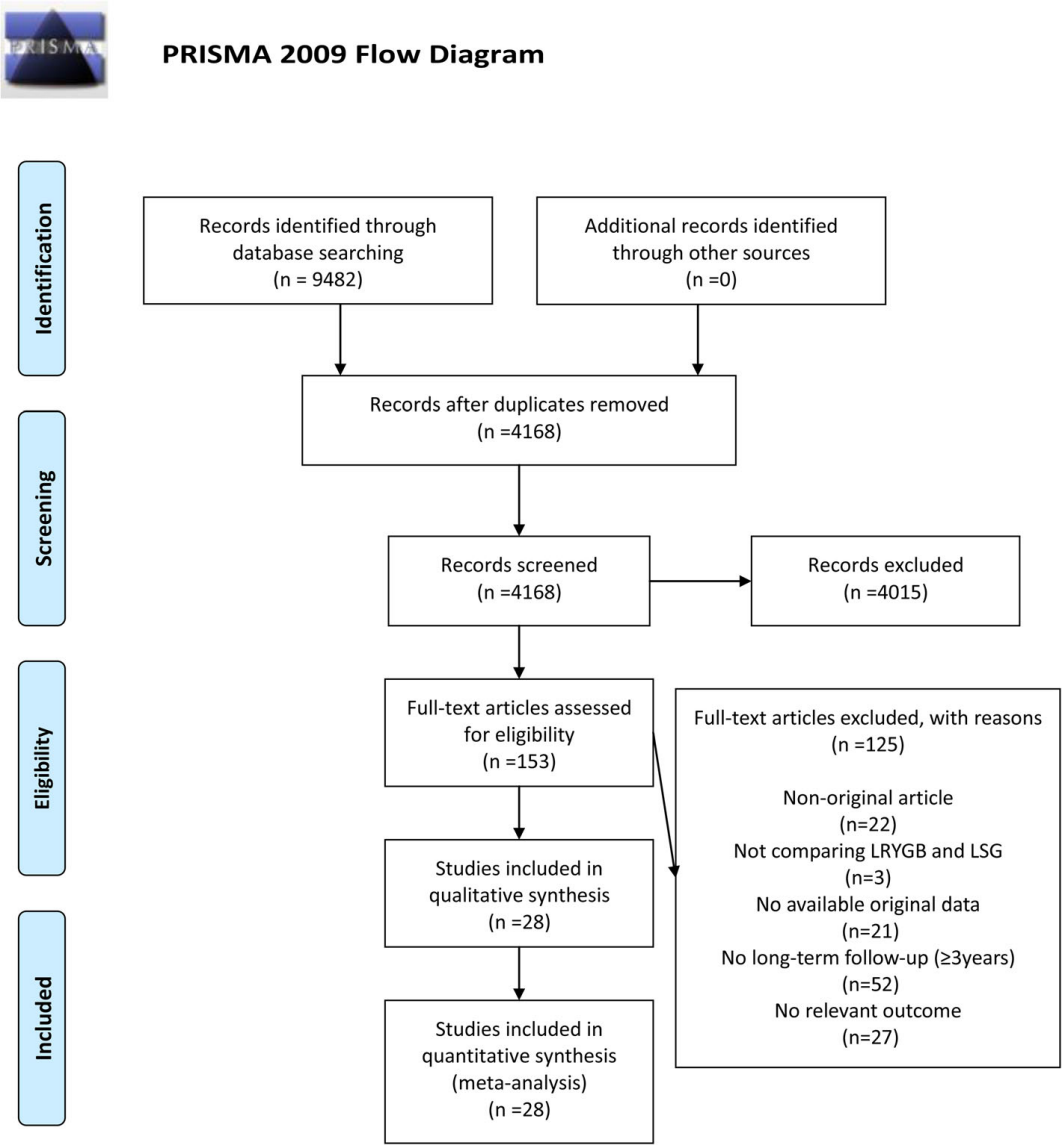
Flow diagram of study inclusion and exclusion

The PRISMA flowchart of literature search is shown in Fig. 1. The initial database search retrieved 9482 articles. No article was found through other sources. After removing 5314 duplicates, 4015 publications for non-surgical procedures were excluded. Among the 153 publications that met our criteria, 3 were excluded as they did not compare LSG and LRYGB, 22 were non-original articles, and 21 had no available original data. The follow-up of 52 studies was less than 3 years, and 27 articles had no outcomes relevant to our bariatric surgery. Therefore, 28 articles were included in analysis [1–7, 10, 11, 14, 15, 17–20, 24–36]. After serious consideration, all articles were of great research value and could provide strong evidence for our meta-analysis. Countries included were very representative and vast, including Chile, China, Finland, France, Greece, India, Italy, Lebanon, Netherlands, Poland, Singapore, Spain, Switzerland, USA, and Venezuela.

### Study characteristics

In total, the characteristics of the 28 studies are shown in Table 1.The included studies consist of seven RCTs, six prospective observational studies, and 15 retrospective observational studies. Overall, 9038 patients (4597 in the LSG group, 4441 in the LRYGB group) were included. All of them were followed for at least 3 years, and 13 studies of them were followed for 5 years or longer. The results of assessment of quality and risk of bias for all included studies were included in Additional file 1: Table S1.

### %EWL

**Fig. 2:**
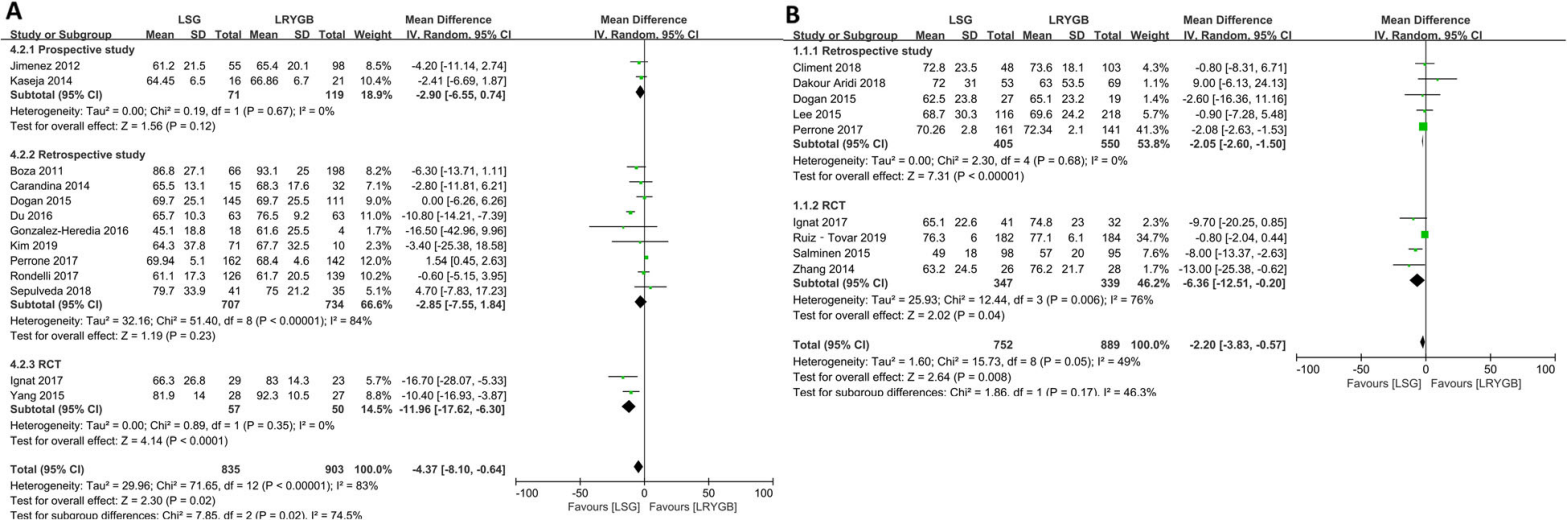
Forest plots of of %EWL (LSG vs LRYGB). (A) Third year and (B) fifth year

%EWL is an essential metric that measures the effect of weight loss after bariatric surgery. A total of 19 articles reported %EWL after surgery. Among them, 13 articles provided 3-year follow-up data, 9 articles provided 5-year follow-up data, and 3 articles provided both 3-year and 5-year data. At 3-year follow-up, %EWL in the LRYGB group was greater than that in the LSG group (WMD = -4.37, 95%Cl = − 8.10-(− 0.64), *p* = 0.02, random-effects model). Subgroup analysis performed according to the type of study revealed that the postoperative effect of LRYGB group was better than that of LSG group in RCTs (WMD = -11.96, 95%Cl = − 17.62-(− 6.30), *p* < 0.001). Moreover, no significant difference in the treatment effect between the two groups was found, in either prospective or retrospective studies (Fig. 2).

Comparison of the outcome of %EWL in the fifth postoperative year showed that patients who underwent LRYGB had greater %EWL than those who underwent LSG (WMD = -2.20, 95%Cl = − 3.83-(− 0.57), *p* = 0.008, random-effects model). Subgroup analysis of LSG and LRYGB in 5-year follow-up revealed that the LRYGB group has better outcomes than the LSG group in both retrospective studies (WMD = -2.05, 95% Cl = − 2.60-(− 1.50), *p* < 0.001) and RCTs (WMD = -6.36, 95% Cl = − 12.51-(− 0.20), *p* = 0.04) (Fig. 2).

### Resolution of obesity-related comorbidities

Many studies investigated the improvement of obesity-related comorbidities during the postoperative period. Some publications discussed comorbidities such as arthritis, obstructive sleep apnea, hyperuricemia, and depression. This meta-analysis detected the effect of remission of only hypertension, T2DM, and dyslipidemia.

### T2DM

**Fig. 3:**
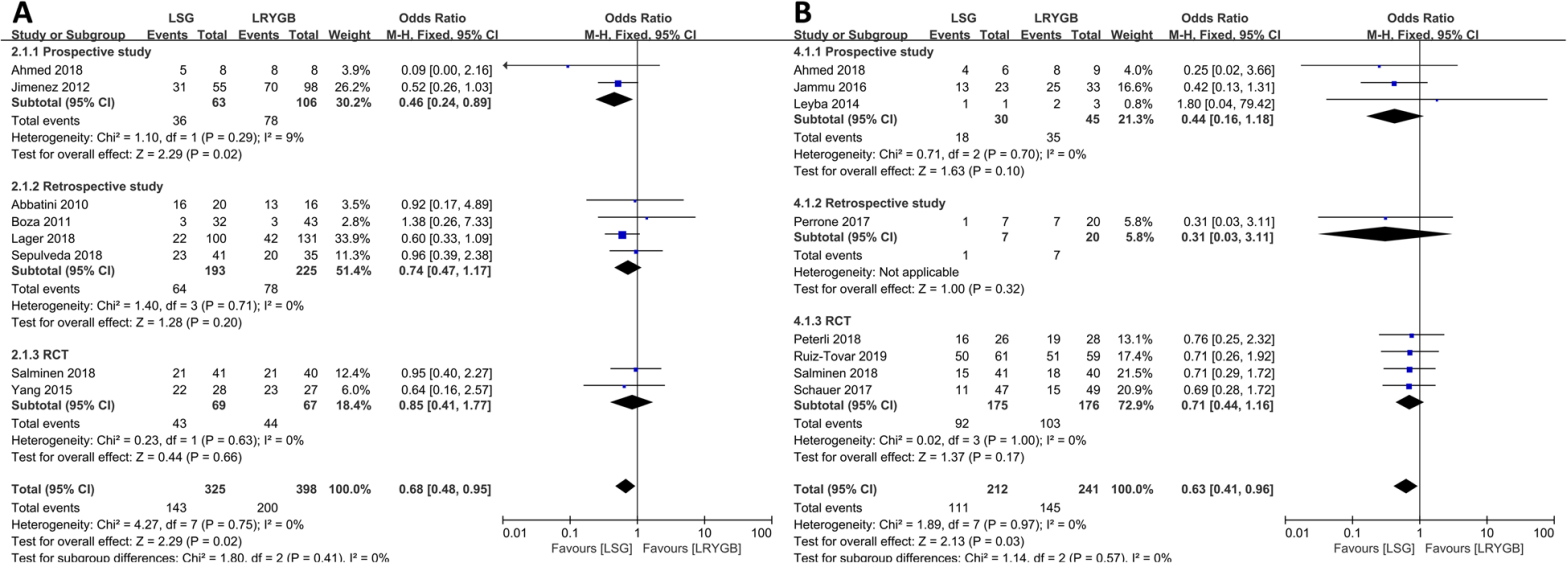
Forest plots of remission of T2DM (LSG vs LRYGB). (A) Third year and (B) fifth year

Of the 28 articles, 14 mentioned remission rates of T2DM representing 1018 patients (490 in the LSG group, 528 in the LRYGB group). The remission rate of T2DM in the LRYGB group was higher than that in the LSG in 3-years follow-up (OR = 0.68, 95% CI = 0.48–0.95, *p* = 0.02, fixed-effects model), so was the remission rate in 5-year follow-up (OR = 0.63, 95% CI = 0.41–0.96, *p* = 0.03, fixed-effects model). Subgroup analysis according to the type of study revealed that the remission rate in LRYGB was higher than that in LSG in prospective studies with 3-year follow-up (OR = 0.46, 95% CI = 0.24–0.89, *p* = 0.02). In addition, no significant difference was found between retrospective studies and RCTs. At 5-year follow-up, no statistical difference was noted among all prospective studies, retrospective studies, or RCTs. Details are shown in Fig. 3.

### Hypertension

**Fig. 4:**
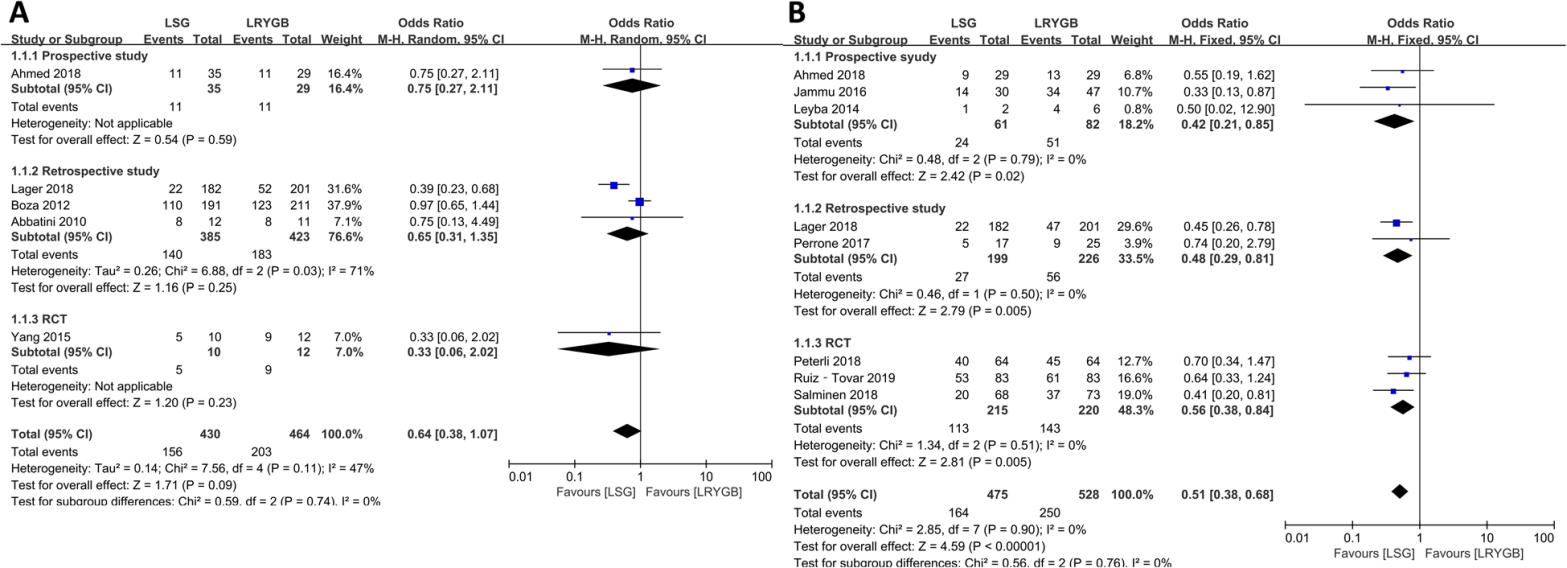
Forest plots of remission of hypertension (LSG vs LRYGB). (A) Third year and (B) fifth year

Eleven articles focused on hypertension representing 1456 patients (694 in the LSG group, 762 in the LRYGB group). No statistical difference was observed in hypertension at the 3-year follow-up and all subgroup analyses between LRYGB and LSG. Interestingly, compared with the LSG group, the LRYGB group has higher remission rate of hypertension during the fifth postoperative year (OR = 0.51, 95% CI = 0.38–0.68, *p* < 0.001, fixed-effects model). In the subgroup analysis, the outcomes of LRYGB were better than those of LSG among all prospective studies, retrospective studies, and RCTs (Fig. 4).

### Dyslipidemia

**Fig. 5:**
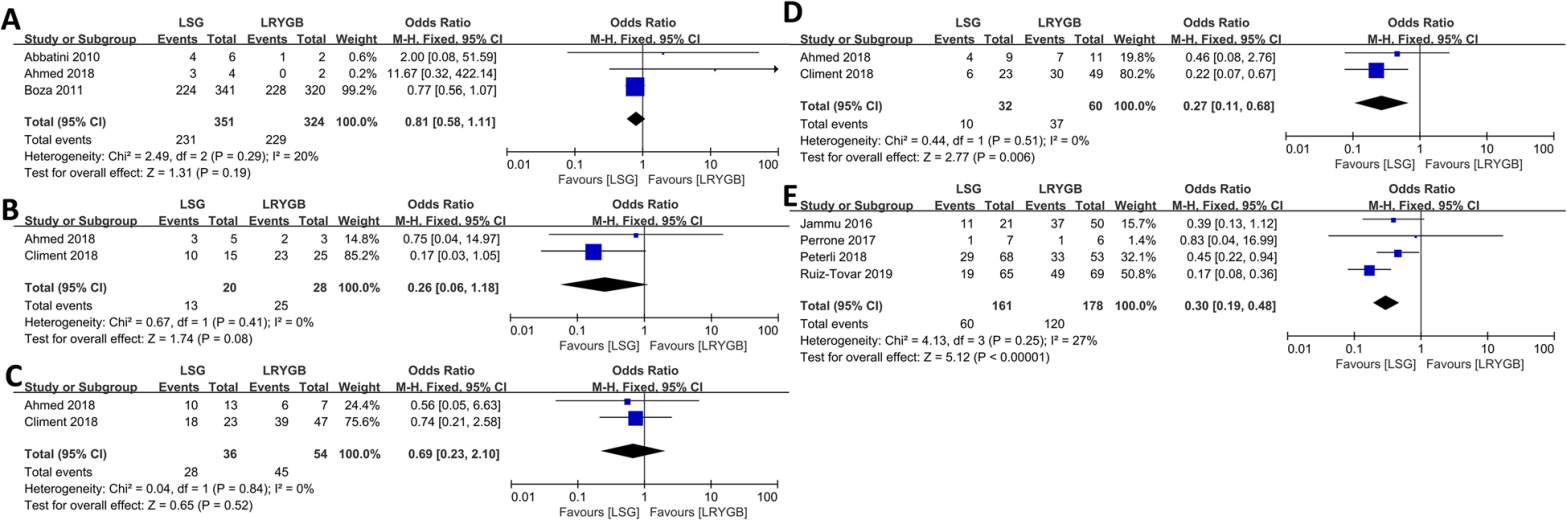
Forest plots of remission of dyslipidemia (LSG vs LRYGB). (A) Remission of dyslipidemia in the third year, (B) remission of high triglycerides in the fifth year, (C) remission of low HDL in the fifth year, (D) remission of high LDL in the fifth year, and (E) remission of dyslipidemia in the fifth year

Although no statistical difference in remission of dyslipidemia at 3-year follow-up was found, LRYGB had higher remission rate of dyslipidemia at 5-year follow-up (OR = 0.30, 95% CI = 0.19–0.48, *p* < 0.001, fixed-effects model). The remission rate of abnormally low-density lipoprotein (LDL) at 5-year follow-up resolved considerably more common in the LRYGB group than in the LSG group (OR = 0.27, 95% CI = 0.11–0.68, *p* = 0.006, fixed-effects model). Moreover, we did not find significant difference in the treatment effect between the two groups in terms of high-density lipoprotein and triglycerides in the fifth year of follow-up (Fig. 5).

### Sensitivity analysis and publication bias

In each group analysis, we excluded each study and the overall effect was consistent. Begg’s and Egger’s test were performed on the results of each group, and the results showed no publication bias (*p* > 0.05).

## Discussion

LSG and LRYGB are the most commonly performed bariatric surgeries in the last decade. They are not only safe and effective for weight loss, but also have a role in alleviating complications. This meta-analysis was based on 27 multi-screened, high-quality highly reviewed articles. The main results of this meta-analysis were as follows: (1) no significant difference was found in %EWL and remission of hypertension and dyslipidemia between the LSG group and the LRYGB group at 3-year follow-up. (2) Five-year follow-up results showed that LRYGB had an advantage over LSG in terms of %EWL, remission of hypertension, dyslipidemia, and abnormally LDL. (3) The remission rate of T2DM was superior in LSG in both 3- and 5-year follow-up.

### %EWL

Our mid-term (3-year follow-up) results showed that both groups had good outcomes and that there was no significant difference in %EWL, which might be attributed to a lower initial body mass index (BMI) [37]. Hosam et al. observed that the preoperative BMI had a strong impact on the final outcome of LSG [38]. Interestingly, LRYGB gradually showed its advantages during the 5-year follow-up period, which agreed with the research conclusions of Zhang et al. [6]. As a restrictive procedure, high-calorie and high-sugar foods after LSG could cause rebound weight gain, so patients should strictly follow the postoperative nutritional guidelines. LSG reduces weight by limiting calories, but expanding sleeved stomach caused by dietary failure will lead to weight recovery. Many articles indicated that LRYGB was superior to LSG in weight reduction especially for super-obese patients [3, 6]. Thus, LSG might be converted to LRYGB due to insufficient weight loss or severe reflux esophagitis [5, 27].

Nowadays, the goal of bariatric surgery is not as simple as controlling weight alone. It is equally important to improve complications such as T2DM, dyslipidemia, hypertension, obstructive sleep apnea, liver, and kidney function damage [39]. Bariatric surgery could also be used as potential treatment of metabolic syndrome, and the concept of “metabolic surgery” was born [40].

### T2DM

The results of the 3-year and 5-year follow-up showed that the T2DM remission rate of LRYGB was higher than that of LSG. The heterogeneity of the results was very low, and data were highly feasible. Jiménez et al. showed that more than half of patients who underwent LSG bariatric surgery had a T2DM remission after 3 years of follow-up, and this goal was achieved with only 2 years in the LRYGB group. T2DM will recur after a long time, which results in decreased remission rate; therefore, long-term follow-up is more meaningful [25]. Some research results showed that %EWL was a determinant of T2DM remission. To determine the effect of fasting insulin and glucose reduction, the degree of %EWL was more significant than the type of surgery. This may be the reason why LRYGB was more effective than LSG as T2DM treatment [16].

Many studies had shown that hormonal changes also play an important role in weight loss and remission of metabolic disease after surgery. Several theories about hormonal changes were intensively under investigation, but none of them currently stand out as the leading mechanism. According to previous studies, LRYGB and LSG induce similar changes in these hormones expect for ghrelin. Ghrelin is reduced after LSG as large parts of the stomach were resected, whereas ghrelin may increase or remain stable after LRYGB [41, 42]. A recent study showed that LRYGB was characterized by accelerated absorption of glucose and amino acids, whereas protein metabolism after LSG did not differ significantly from controls, suggesting that different mechanisms explain improved glycemic control and weight loss after these surgical procedures [43].

### Hypertension

Obesity has become one of the most important causes of hypertension, as 60 to 70% of hypertension in adults can be attributed to obesity [44]. In this study, no statistical difference in midterm hypertension remission rates was found between the two groups, but long-term results showed that LRYGB had an obvious advantage. The results of these studies were similar to our results [2, 10, 15, 20]. The exact mechanism of hypertension and a range of cardiovascular diseases due to obesity have not been confirmed. However, the neuroendocrine system and adipokines were thought to play a leading role. Obesity-related hypertension was also thought to be associated with metabolic syndrome related to glucose intolerance [44, 45]. Previous studies had shown that LRYGB may be the first choice for obese patients with cardiovascular risk [46].

### Dyslipidemia

Compared to LSG, LRYGB was a more effective treatment of dyslipidemia [37], but the two groups had similar effects on obesity metabolic disorders [17]. Studies that combined various factors showed that the remission rate of abnormally LDL in LRYGB was higher than that in LSG [27, 32], because LRYGB might have reduced the absorption rate of LDL than did LSG [32]. Other studies have shown that LSG has no significant effect on the reduction of LDL, while LRYGB could cause absorption disorder in individuals; perhaps, this is the reason why LRYGB could improve the overall lipid profile better than LSG [47].

The advantages of LRYGB were as follows: The short-term results of LRYGB might be as effective as those of LSG, but medium- or long-term results could show a clear advantage. LRYGB could ensure a good quality of life and minimum side effects, effectively control metabolic complications including T2DM and hypertension [3, 15], provide better blood glucose control, and lower blood lipid level effect [5].

The advantages of the study were a large population base, detailed subgroup analysis (based on follow-up time divided into 3 years (mid-term) and 5 years (long term), three types of trials, and clear indicators for recovery of complications (included only in remission). In a retrospective review of similar meta-analyses, results of Yang et al.’s meta-analysis were similar to the results of the present study in that LRYGB patients had significantly reduced their weight during the 3- or 5-year follow-up period compared to LSG patients [48]. However, they combined the improvement and remission of complications. Heterogeneity could be introduced because of the different definitions of improvement and remission. This might be the reason why LRYGB had not shown an advantage in treating comorbidities. Huang et al.’s meta-analysis showed that LSG and LRYGB had no significant advantage in short- or long-term blood glucose control, which is contrary to our conclusion, because their patients were followed at 1–2 years, and there are limited studies with 3- or 5-year follow-up periods. In addition, they did not perform subgroup analyses based on the type of trial, which was less convincing than this article [49].

Zhao et al. [50]. included 11 RCTs in their meta-analysis, and the results showed no difference in %EWL and T2DM between the two surgical methods (LSG and LRYGB), which may be attributed to the lack of long-term follow-up data, with only 3 studies providing data of 3 years and 5 years respectively. Sharples [51] included 5 RCTs and concluded demonstrated a significantly greater %EWL in patients undergoing LRYGB compared with LSG. However, there was no significant difference between LRYGB and LSG in rates of resolution or improvement of diabetes. Similarly, HbA1C levels were not significantly different between the two procedures. A meta-analysis involving 33 RCTs showed that LRYGB resulted in greater BMI loss at 1 and 3 years; however, there was insufficient randomized evidence to draw any conclusions regarding weight loss between the 2 procedures at 5 years. No differences between the two procedures were found in remission of type 2 diabetes, despite a trend at every time interval favoring LRYGB, hypertension [52]. However, most of the studies included in this study were short-term follow-up data with high heterogeneity, so the results need careful interpretation.

This study has some limitations. This study included retrospective studies that reduced the overall quality of evidence. We were unable to classify obese people according to the degree of obesity, because it was not explicitly mentioned in the papers. In addition, most of the countries included were Western and were not necessarily suitable for the Asian population. Finally, the criteria for incorporating obese patients were different because varied literature types were included. These deficiencies will affect the scope and accuracy of the findings of this meta-analysis.

## Conclusion

In summary in terms of the long-term effects of bariatric surgery, including %EWL and the remission of complications (T2DM, hypertension and dyslipidemia), the effect of LRYGB was better than of LSG.

## Additional File

**Additional file 1 Table S1** Quality assessment of studies included.

## Data Availability

All data generated or analysed during this study are included in this published article.

## Abbreviations

BMI: Body mass index
EWL: Percentage of excess weight loss
LDL: Low-density lipoprotein, 95%CI: 95% confidence intervals
LRYGB: Laparoscopic Roux-en-Y gastric bypass
LSG: Laparoscopic sleeve gastrectomy
NOS: Newcastle-Ottawa Quality Assessment Scale
OR: Odds ratio
PRISMA: Preferred Reporting Items for Systematic Reviews and Meta-Analyses, %
RCT: Randomized controlled trial
T2DM: Type 2 diabetes mellitus
WMD: Weighted mean difference
