# Supplementary material for: A meta-analysis of the medium- and long-term effects of laparoscopic sleeve gastrectomy and laparoscopic Roux-en-Y gastric bypass
Source: BMC Surg. 2020 Feb 12;20:30. doi: 10.1186/s12893-020-00695-x (PMC7014764; doi:10.1186/s12893-020-00695-x)
Supplement: Supplementary file 1 — Additional file 1 Table S1 Quality assessment of studies included. [file 12893_2020_695_MOESM1_ESM.docx]

**Supplementary Table1.** Quality assessment of studies included.

| Author, year,  Study (RCT) | Sequence  Generation | | Allocation  Concealment | | Blinding | Incomplete  outcome data | | Selective  outcome reporting | | Free of  other bias |  |  |  |  |  |
| --- | --- | --- | --- | --- | --- | --- | --- | --- | --- | --- | --- | --- | --- | --- | --- |
| Ignat, 2017 | Low risk | | Low risk | | Unclear risk | Low risk | | Low risk | | Low risk |  |  |  |  |  |
| Peterli, 2018 | Low risk | | Low risk | | High risk | Low risk | | Low risk | | Low risk |  |  |  |  |  |
| Ruiz-Tovar, 2019 | Low risk | | Unclear risk | | Low risk | Low risk | | Low risk | | Low risk |  |  |  |  |  |
| Salminen, 2018 | Unclear risk | | Unclear risk | | Low risk | Low risk | | Low risk | | Low risk |  |  |  |  |  |
| Yang, 2015 | Low risk | | High risk | | Low risk | Low risk | | Unclear risk | | Low risk |  |  |  |  |  |
| Zhang, 2014 | Low risk | | Low risk | | Unclear risk | High risk | | Low risk | | Low risk |  |  |  |  |  |
| Schauer, 2017 | Low risk | | Unclear risk | | High risk | Low risk | | Low risk | | Low risk |  |  |  |  |  |
| Author, year,  Study (Observational) | | **Selection (Out of 4)** | | | | | | | | | **Comparability**  **(Out of 2)** | **Outcomes(Out of 3)** | | | **Total**  **(Out of 9)** |
|  |  | Representativeness of exposed cohort | | Selection of nonexposed cohort | | | Ascertainment  of exposure | | Outcome not present at the start of the study | |  | Assessment of outcomes | Length of follow-up | Adequacy of follow-up |  |
| Abbatini, 2010 | | 0 | | 0 | | | 1 | | 0 | | 2 | 1 | 1 | 1 | 6 |
| Ahmed, 2018 | | 1 | | 1 | | | 1 | | 1 | | 2 | 1 | 1 | 1 | 9 |
| Alexandrou, 2014 | | 1 | | 1 | | | 1 | | 1 | | 2 | 0 | 1 | 1 | 8 |
| Dakour Aridi, 2018 | | 1 | | 1 | | | 1 | | 0 | | 1 | 1 | 1 | 1 | 7 |
| Boza, 2012 | | 1 | | 0 | | | 1 | | 1 | | 2 | 1 | 1 | 0 | 8 |
| Carandina, 2014 | | 1 | | 1 | | | 1 | | 0 | | 2 | 1 | 0 | 1 | 7 |
| Dogan, 2015 | | 1 | | 1 | | | 1 | | 0 | | 1 | 1 | 1 | 1 | 7 |
| Du, 2016 | | 1 | | 1 | | | 1 | | 1 | | 1 | 1 | 1 | 0 | 7 |
| Climent, 2018 | | 1 | | 1 | | | 1 | | 1 | | 1 | 1 | 1 | 1 | 8 |
| Gonzalez-Heredia, 2016 | | 1 | | 1 | | | 1 | | 0 | | 1 | 1 | 1 | 0 | 6 |
| Jammu, 2016 | | 1 | | 1 | | | 1 | | 0 | | 2 | 1 | 1 | 0 | 7 |
| Jimenez, 2012 | | 1 | | 1 | | | 1 | | 0 | | 1 | 1 | 1 | 0 | 6 |
| Kim, 2019 | | 1 | | 1 | | | 1 | | 1 | | 1 | 1 | 1 | 1 | 8 |
| Kaseja, 2014 | | 1 | | 1 | | | 1 | | 0 | | 2 | 1 | 1 | 1 | 8 |
| Lager, 2018 | | 1 | | 1 | | | 1 | | 0 | | 1 | 1 | 1 | 1 | 7 |
| Lee, 2015 | | 1 | | 1 | | | 1 | | 1 | | 2 | 0 | 1 | 1 | 8 |
| Leyba, 2014 | | 1 | | 1 | | | 1 | | 0 | | 2 | 1 | 1 | 1 | 8 |
| Perrone, 2017 | | 1 | | 1 | | | 1 | | 0 | | 1 | 1 | 1 | 1 | 7 |
| Rondelli, 2017 | | 1 | | 1 | | | 1 | | 0 | | 1 | 1 | 1 | 1 | 7 |
| Sepulveda, 2018 | | 1 | | 1 | | | 1 | | 0 | | 2 | 1 | 1 | 1 | 8 |
| Vidal, 2013 | | 1 | | 1 | | | 1 | | 0 | | 1 | 1 | 1 | 1 | 7 |

The RCTs and observational studies were assessed by the Cochrane Collaboration’s tool and Newcastle-Ottawa Quality Assessment Scale, respectively.

Risk of bias was assessed as “low risk”, “high risk” or “unclear risk”.
